# Quantification, Exchange, and Removal of Surface Ligands on Noble-Metal Nanocrystals

**DOI:** 10.1021/acs.accounts.3c00116

**Published:** 2023-05-10

**Authors:** Kei Kwan Li, Chia-Ying Wu, Tung-Han Yang, Dong Qin, Younan Xia

**Affiliations:** † School of Materials Science and Engineering, Georgia Institute of Technology, Atlanta, Georgia 30332, United States; ‡ The Wallace H. Coulter Department of Biomedical Engineering, Georgia Institute of Technology and Emory University, Atlanta, Georgia 30332, United States; § School of Chemistry and Biochemistry, Georgia Institute of Technology, Atlanta, Georgia 30332, United States; ⊥ Department of Chemical Engineering, National Tsing Hua University, Hsinchu 30013, Taiwan

## Abstract

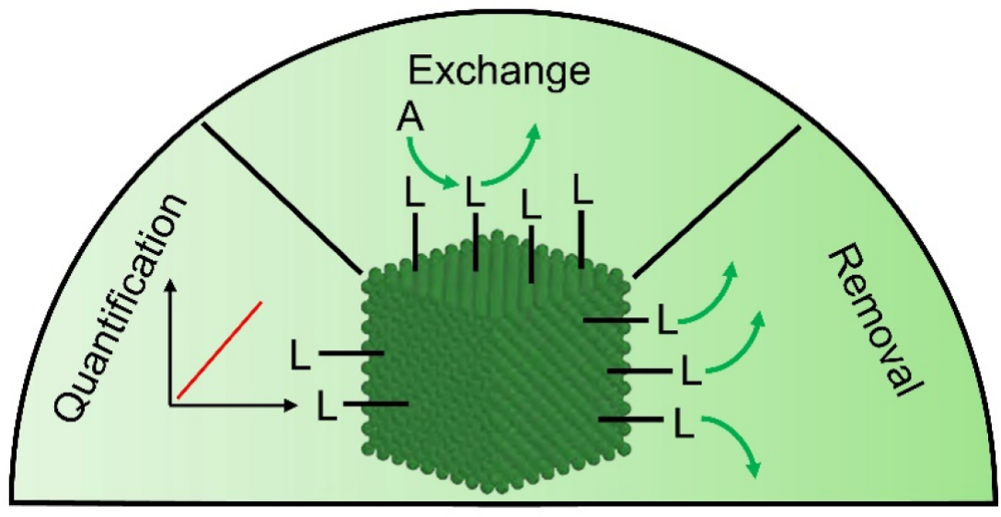

Surface ligands are vital to the colloidal synthesis of noble-metal nanocrystals with well-controlled sizes and shapes for various applications. The surface ligands not only dictate the formation of nanocrystals with diverse shapes but also serve as a colloidal stabilizer to prevent the suspended nanocrystals from aggregation during their synthesis or storage. By leveraging the facet selectivity of some surface ligands, one can further control the sites for growth or galvanic replacement to transform presynthesized nanocrystals into complex structures that are otherwise difficult to fabricate using conventional methods. Furthermore, the presence of surface ligands on nanocrystals also facilitates their applications in areas such as sensing, imaging, nanomedicine, and self-assembly. Despite their popular use in enhancing the properties of nanocrystals and thus optimizing their performance in a wide variety of applications, it remains a major challenge to quantitatively determine the coverage density of ligand molecules, not to mention the difficulty of substituting or removing them without compromising the surface structure and aggregation state of the nanocrystals.

In this Account, we recapitulate our efforts in developing methods capable of qualitatively or quantitatively measuring, exchanging, and removing the surface ligands adsorbed on noble-metal nanocrystals. We begin with an introduction to the typical interactions between ligand molecules and surface atoms, followed by a discussion of the Langmuir model that can be used to describe the adsorption of surface ligands. It is also emphasized that the adsorption process may become very complex in the case of a polymeric ligand due to the variations in binding configuration and chain conformation. We then highlight the capabilities of various spectroscopy methods to analyze the adsorbed ligands qualitatively or quantitatively. Specifically, surface-enhanced Raman scattering, Fourier transform infrared, and X-ray photoelectron spectroscopy are three examples of qualitative methods that can be used to confirm the absence or presence of a surface ligand. On the other hand, ultraviolet–visible spectroscopy and inductively coupled plasma mass spectrometry can be used for quantitative measurements. Additionally, the coverage density of a ligand can be derived by analyzing the morphological changes during nanocrystal growth. We then discuss how the ligands present on the surface of metal nanocrystals can be exchanged directly or indirectly to meet the requirements of different applications. The former can be done using a ligand with stronger binding, whereas the latter is achieved by introducing a sacrificial shell to the surface of the nanocrystals. Furthermore, we highlight three additional strategies besides simple washing to remove the surface ligands, including calcination, heating in a solution, and UV-ozone treatment. Finally, we showcase three applications of metal nanocrystals in nanomedicine, tumor targeting, and self-assembly by taking advantage of the diversity of surface ligands bearing different functional groups. We also offer perspectives on the challenges and opportunities in realizing the full potential of surface ligands.

## Key References

- YangT.-H.; AhnJ.; ShiS.; QinD.Understanding the Role of Poly(vinylpyrrolidone) in Stabilizing and Capping Colloidal Silver Nanocrystals. ACS Nano2021, 15, 14242–1425210.1021/acsnano.1c01668.34436857(1)*This work used surface-enhanced Raman spectroscopy to reveal the conformations of poly(vinylpyrrolidone) adsorbed on Ag nanocubes when dispersed in different solvents.*
- ZhouS.; HuoD.; GoinesS.; YangT.-H.; LyuZ.; ZhaoM.; GilroyK. D.; WuY.; HoodZ. D.; XieM.; XiaY.Enabling Complete Ligand Exchange on the Surface of Gold Nanocrystals through the Deposition and Then Etching of Silver. J. Am. Chem. Soc.2018, 140, 11898–1190110.1021/jacs.8b06464.30179474(2)*An indirect method was validated for removing cetyltrimethylammonium chloride from the surface of Au nanocrystals through the deposition of an ultrathin layer of Ag, followed by selective etching of the Ag in the presence of a new ligand such as citrate or PVP.*
- PengH.-C.; XieS.; ParkJ.; XiaX.; XiaY.Quantitative Analysis of the Coverage Sensity of Br^–^ Ions on Pd{100} Facets and Its Role in Controlling the Shape of Pd Nanocrystals. J. Am. Chem. Soc.2013, 135, 3780–378310.1021/ja400301k.23438500(3)*Inductively coupled plasma mass spectrometry was adapted to quantify the amounts of Br*^*–*^*chemisorbed on Pd nanocrystals with different shapes.*
- XiaX.; ZengJ.; OetjenL. K.; LiQ.; XiaY.Quantitative Analysis of the Role Played by Poly(vinylpyrrolidone) in Seed-Mediated Growth of Ag Nanocrystals. J. Am. Chem. Soc.2012, 134, 1793–180110.1021/ja210047e.22206387PMC3274755(4)*This work illustrated how to derive the coverage density of PVP on Ag nanocubes by following the growth of Ag cubic seeds in the presence of PVP at different concentrations.*

## 1 Introduction

**Figure 1:**
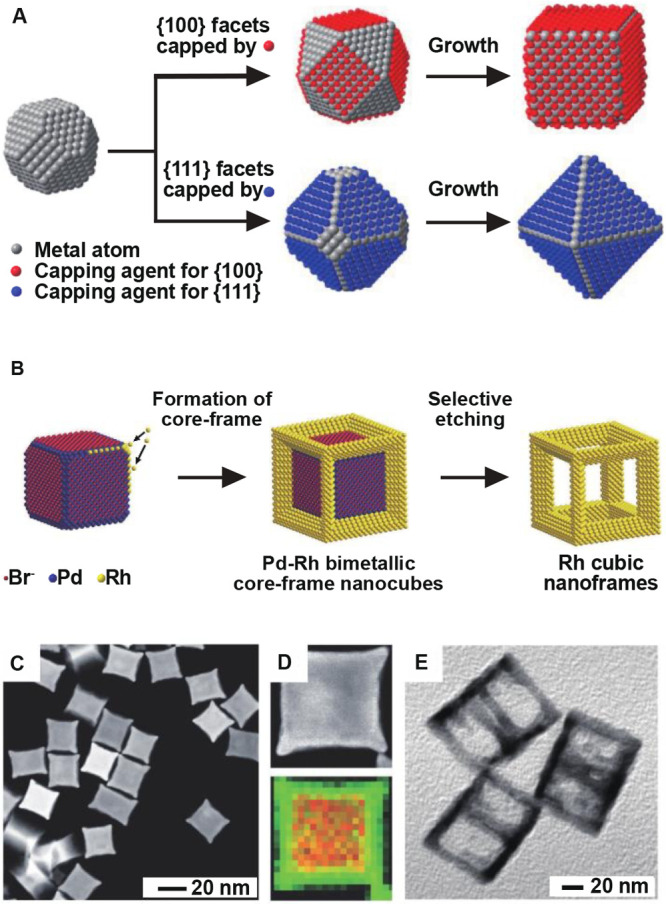
(A) Schematic illustrating the role of surface ligands in directing the growth of single-crystal seeds into nanocrystals with different shapes. Reproduced with permission from ref (6). Copyright 2015 American Chemical Society. (B) Schematic illustrating the synthesis of Rh nanoframes by capping the {100} facets of Pd nanocubes with bromide. (C, D) TEM images and energy-dispersive X-ray (EDX) mapping of Pd@Rh core-frame nanocubes (red = Pd, green = Rh). (E) TEM image of Rh nanoframes. Reproduced with permission from ref (10). Copyright 2012 VCH-Wiley.

Surface ligands offer a versatile handle to functionalize nanocrystals for applications in medicine, including tumor targeting and drug delivery. They are also essential to the synthesis of colloidal nanocrystals with well-controlled sizes and shapes for applications in catalysis, photonics, and electronics.^5^ The mechanism for shape control can be thermodynamic or kinetic in nature.^6−8^ In terms of thermodynamics, the ligands selectively cap a specific type of facet to alter the surface free energy landscape and thus control the shape taken by nanocrystals during their growth. An example can be found in the synthesis of Ag nanocrystals with a cubic or octahedral shape from the same type of single-crystal seed, as illustrated in Figure 1A.^6^ In the presence of a capping ligand for {100} facets, the atoms prefer to grow from {111} facets, resulting in products with a cubic shape enclosed by {100} facets. On the other hand, an octahedral nanocrystal is formed when the ligand caps {111} facets. In terms of kinetics, the capping agent is thought to affect the flux of atoms, with a stronger binding of poly(vinylpyrrolidone) (PVP) toward Ag(100) leading to greater flux of atoms to {111} facets and thus the formation of nanocubes encased by {100} facets.^8^ Enabled by facet-selective ligands, noble-metal nanocrystals have been synthesized with diverse shapes and thus different catalytic activities toward various reactions.^9^

The facet selectivity of surface ligands can also be utilized to achieve site-selected overgrowth on preformed nanocrystals, offering a versatile route to complex structures with unique properties. A notable example is the synthesis of Pd@Rh core–frame nanocubes and their subsequent conversion to Rh nanoframes by selectively etching away the Pd cores, as shown in Figure 1B.^10^ Since the Pd{100} facets are capped by Br^–^, Rh atoms are forced to nucleate from the corners and edges to generate a frame. Site-selected galvanic replacement has also been achieved through the introduction of a proper surface ligand,^11^ in which the introduction of Br^–^ promoted the galvanic reaction between Pd nanocubes and Pt(IV) precursor to synthesize Pd–Pt bimetallic nanocubes with a highly concaved surface.

In this Account, we begin with an introduction to the typical interactions between ligands and surface atoms. We then discuss recent progress in quantifying, substituting, and removing the surface ligands adsorbed on noble-metal nanocrystals. Finally, we illustrate how the ligand on the surface of nanocrystals can be engineered to enable or enhance applications in nanomedicine, tumor targeting, and self-assembly.

## 2 Interaction between Ligand and Surface

The interaction between ligand and surface may and may not involve chemical bonding, corresponding to the situations of chemisorption and physisorption, respectively. Physisorption occurs when the ligand is immobilized on a surface through relatively weak forces such as van der Waals and/or dipole interactions. Chemisorption, in contrast, involves a much stronger interaction, such as metal–ligand coordinate bonding. Regardless of the interaction involved, the adsorption of small-molecule ligands can be described using the Langmuir model. At equilibrium, the fractional surface coverage (θ) of a ligand can be expressed as θ = *K·C*_*bulk*_/(1 + *K·C*_*bulk*_), where *K* is the binding constant, and *C*_*bulk*_ is the concentration of free ligand in the solution. This simple model only applies to a homogeneous surface where the adsorbed molecules do not dissociate, and there is no lateral interaction between adjacent adsorbates.^12^ From the equation, the surface adsorption of a ligand is determined by its affinity toward surface atoms(s) and its concentration in the solution. In general, surface adsorption will become more complex in the case of a polymeric ligand due to its diverse binding configurations and conformations. According to the Flory–Huggins model,^13^ configuration entropy generally decreases when a polymer chain adsorbs onto a solid surface. This must be compensated by adsorption enthalpy from the interaction between the polymer and the surface. As such, advanced models are being developed to explain the dependences of adsorption on particle size and shape. In one study, a population balance model was developed to reveal size-dependent ligand coverage during the formation of Pd nanoparticles.^14^ The larger nanoparticles had a higher ligand coverage than those newly formed, smaller ones and, thus, a slower growth rate.

**Figure 2:**
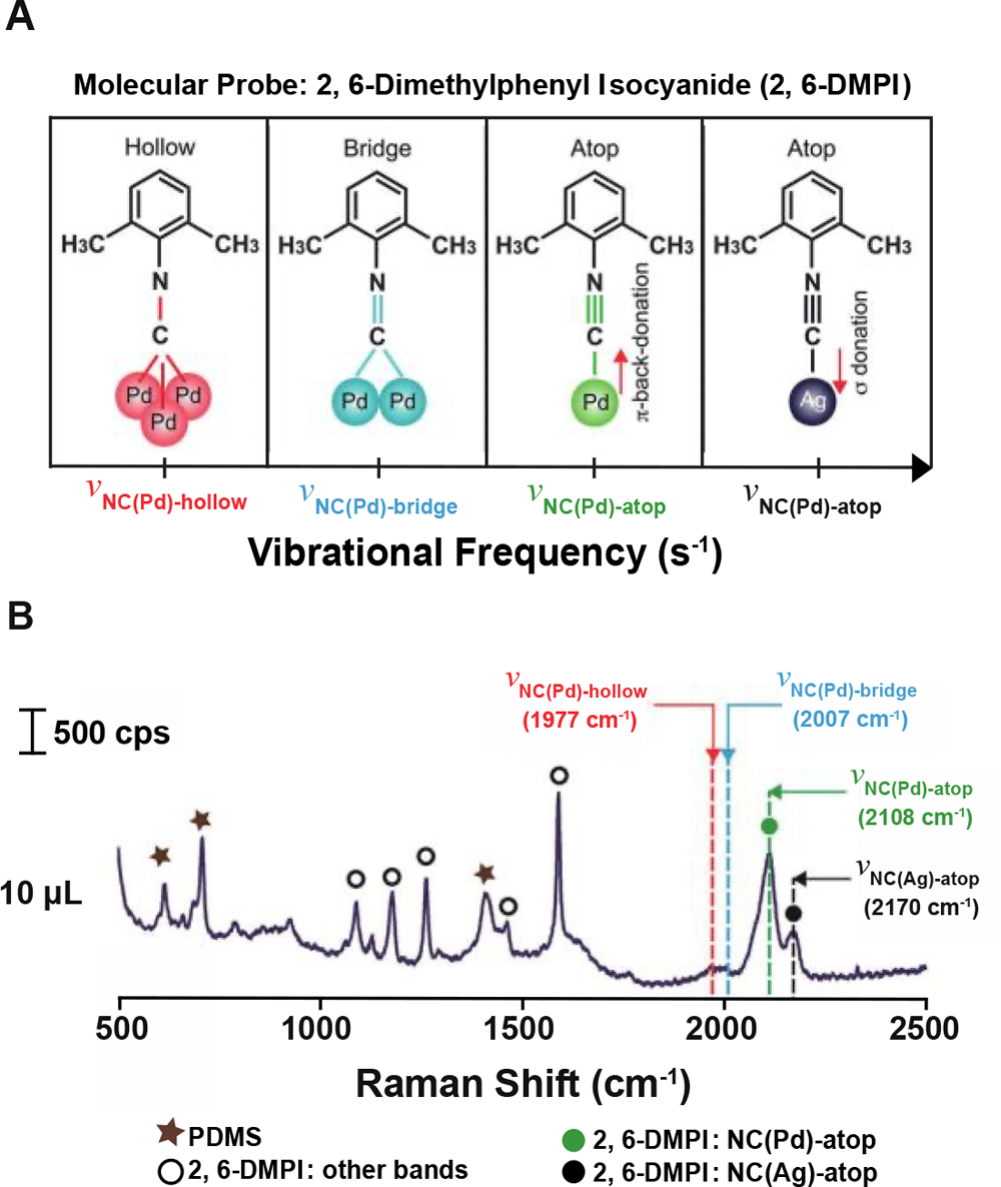
(A) Difference in stretching frequency for the NC bond, ν_NC_, when 2,6-DMPI binds to Ag in the atop configuration and to Pd in the atop, bridge, and hollow configurations, respectively. (B) SERS spectra showing the peaks corresponding to the binding configurations in (A). Reproduced with permission from ref (15). Copyright 2018 American Chemical Society.

We have probed the metal–ligand interaction using spectroscopy methods such as surface enhanced Raman spectroscopy (SERS). Figure 2A shows an example that involved the use of 2,6-dimethylphenyl isocyanide (2,6-DMPI) to track the overgrowth of Pd from the edges of Ag nanocubes *in situ*.^15^ When the isocyanide group (−NC) interacts with Ag and Pd atoms, the difference in binding configuration leads to distinct stretching frequencies for the NC bond (Figure 2B). Specifically, the isocyanide group binds to Ag atoms only through σ donation, strengthening the NC bond and resulting in a blue-shift for ν_NC(Ag)-atop_. In contrast, π-back-donation from Pd would weaken the NC bond, causing a red-shift to ν_NC(Pd)-atop_. Interestingly, the NC bond was progressively weakened as the carbon atom interacted with more Pd atoms in bridge and hollow configurations. Therefore, both ν_NC(Pd)-bridge_ and ν_NC(Pd)-hollow_ were further red-shifted relative to ν_NC(Pd)-atop_. Altogether, SERS can serve as a powerful tool to reveal the interaction between ligand molecules and surface atoms.

## 3 Quantification of Surface Ligand

Knowing the coverage density of a ligand on the surface of metal nanocrystals is essential. This information can be obtained qualitatively or quantitatively, depending on the methods. Generally, spectroscopic methods such as SERS, Fourier transform infrared (FT-IR), X-ray photoelectron spectroscopy (XPS) only provide a qualitative measure, while fluorescence spectroscopy is capable of semiquantitative analysis. On the other hand, ultraviolet–visible (UV–vis) spectroscopy and inductively coupled plasma mass spectrometry (ICP-MS) can be used to obtain quantitative information for some types of ligands. In addition, the coverage density can be derived by monitoring the shape evolution of nanocrystals during seed-mediated growth. In general, each technique only works for certain types of ligands, including halides, polymers, and organic compounds.

### 3.1 Qualitative Measurement

**Figure 3:**
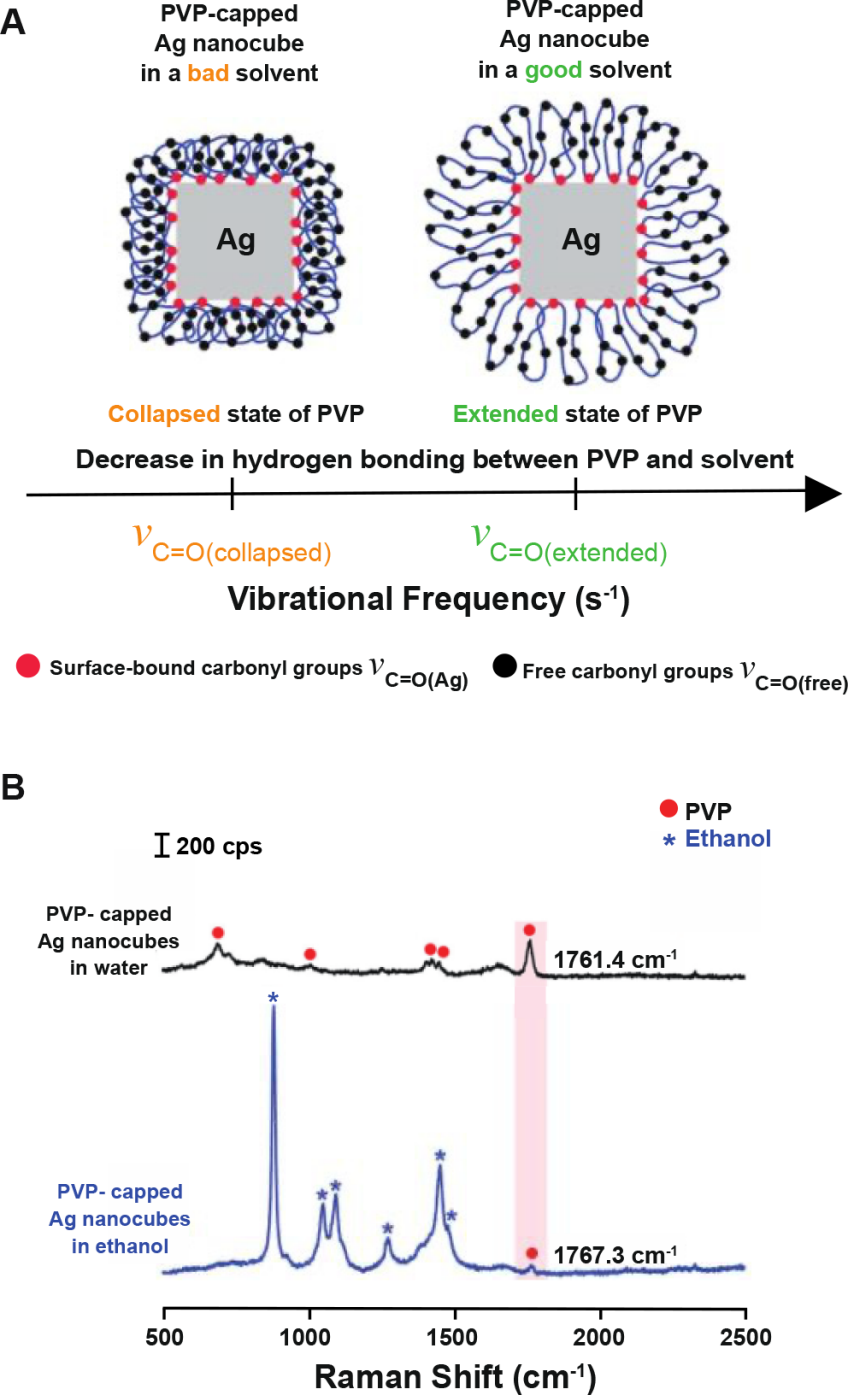
(A) Vibrational frequencies of the ν_C=O_ band of PVP when the nanocubes are dispersed in bad and good solvents, respectively. (B) SERS spectra recorded from PVP-capped Ag nanocubes when they are dispersed in water (bad solvent) and ethanol (good solvent), respectively. Reproduced with permission from ref (1). Copyright 2021 American Chemical Society.

Qualitative measurement only tells the absence or presence of a ligand, with no knowledge of its coverage density. In one study, we used SERS to confirm the presence of PVP on Ag nanocubes by resolving the vibrational peak of its carbonyl group at 1760 cm^–1^.^16^ The peak intensity decreased when the PVP was replaced by cysteamine or thiol-terminated poly(ethylene glycol) (PEG). In another study, we used this peak to analyze the interactions between the adsorbed PVP and the solvent.^1^ As illustrated in Figure 3A, PVP binds to the surface of a Ag nanocube through some of its carbonyl groups, while the segments between adjacent binding sites are expelled into the solvent as loops. The carbonyl groups residing on the loops (ν_C=O(free)_) shifted toward lower frequencies as the hydrogen bonding between PVP and the solvent increased, while that of the carbonyl groups coordinated to the surface (ν_C=O(Ag)_) remained unchanged. As shown in Figure 3B, the SERS peaks were located at 1761.4 and 1767.3 cm^–1^, respectively, when PVP-capped Ag nanocubes were dispersed in water and ethanol. Besides, the PVP loops underwent conformational changes between collapsed and extended states in bad and good solvents, respectively, altering the separation between the free carbonyl groups and the Ag surface and thereby the intensity of the ν_C=O_ peak. Indeed, the expansion of PVP in ethanol led to a significant drop in peak intensity. The SERS results were consistent with the dynamic light scattering (DLS) data. Based on DLS, the hydrodynamic sizes of Ag nanocubes with an edge length of 39.7 ± 1.4 nm were 74.1 ± 1.1 and 95.8 ± 1.4 nm, respectively, in water and ethanol, revealing that the PVP loops were extended as the quality of the solvent was improved.

One can also use FT-IR to confirm the presence of a surface ligand by tracking the vibrational peaks of its functional groups. We have used it to validate the presence of cetyltrimethylammonium chloride (CTAC) and citrate on Au nanospheres.^2^ The former had an IR peak at 2850 cm^–1^, corresponding to the CH_2_ group, while the latter had a peak at 1400 cm^–1^ for the carboxylic group. In general, FT-IR cannot be used to quantify the coverage density of surface ligands as the measurement typically involves attenuated total reflectance, making it difficult to know exactly how many particles are probed. Nevertheless, it has been used to derive the coverage density of PEG on Au nanoparticles with the assistance of a calibration curve.^17^

Halides can be easily detected using conventional techniques such as XPS and EDX. We have used XPS to validate the adsorption of Br^–^on Pd nanocrystals in several studies,^3,18^ where the Br 3d peak was found to appear around 69 eV, in agreement with literature. Sometimes, XPS can also be used to characterize organic ligands such as oleylamine (OAm) due to the presence of nitrogen atoms. In one study,^19^ the adsorbed OAm showed a noticeable N 1s peak at 398 eV. Other groups have reported the use of EDX mapping. Since the ligands were distributed on the surface of a nanocrystal, the mapping data gives a pseudo-core−shell structure, as shown for the Cl^–^ on Ag nanocubes.^20^ In general, XPS is only good for qualitative analysis as it is often difficult to know exactly how many particles are probed. In one study, the coverage density of ligands was derived by analyzing the ratio between the signals from surface atoms and ligand molecules with the help of simulation.^21^ However, the experimental data started to deviate from the simulated value when the particle size was increased beyond 10 nm.

### 3.2 Quantitative Measurement

**Figure 4:**
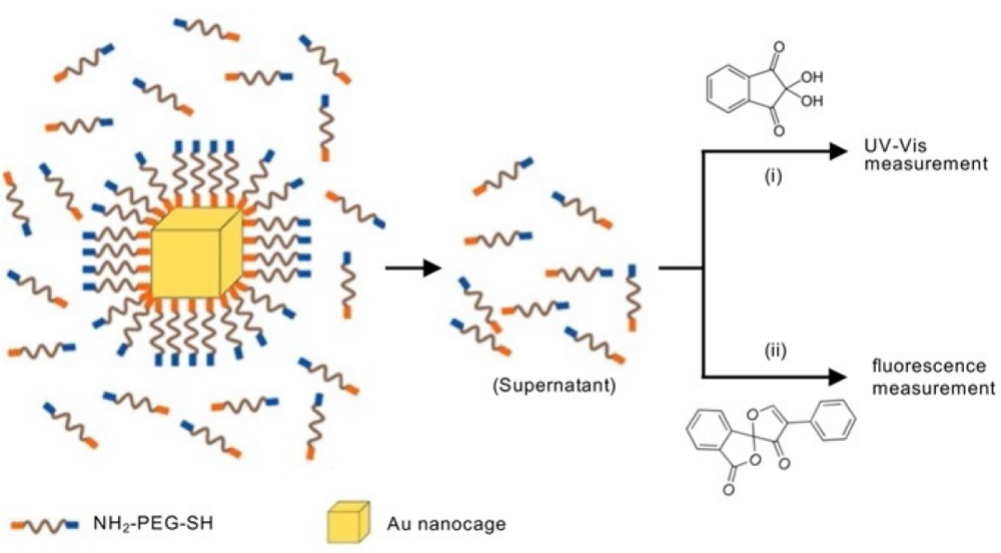
Schematic illustrating two methods for quantifying the average number of NH_2_-PEG-S- per Au nanocage: (i) ninhydrin-based UV–vis absorption and (ii) fluorescamine-based fluorescence assays. Reproduced with permission from ref (22). Copyright 2012 American Chemical Society.

Quantitative measurement is more valuable but can only be achieved in some exceptional cases. For instance, if the surface ligand has strong absorption in the UV–vis region, its total amount on the surface of a known number of nanocrystals can be measured using UV–vis spectroscopy. In some cases, the ligand does not have strong UV–vis absorption, but it can be coupled to a chromophore with strong absorption in the visible region. For example, the -NH_2_ group in NH_2_-PEG-S- can react with ninhydrin to acquire UV–vis absorption. We have applied this method to quantify the number of PEG chains per Au nanoparticle, as shown in Figure 4.^22^ We incubated Au nanoparticles with a known amount of NH_2_-PEG_5000_-SH (molecular weight: 5000) at 4 °C. The supernatant was collected, and the unreacted NH_2_-PEG_5000_-SH was conjugated with ninhydrin for UV–vis quantification. The number of NH_2_-PEG_5000_-S- attached to the Au surface was derived from the difference between the added and remaining quantities. For Au nanocages of 50 nm in size, the average number of -S-PEG_5000_-ninhydrin was 20000 ± 2400, corresponding to a coverage density of 1.33 nm^–2^. We also demonstrated fluorescence measurement by switching from ninhydrin to fluorescamine. Because of its sensitivity to the collection method and the possible involvement of metal-based enhancement, fluorescence can only be semiquantitative. Note that the actual number of ligands should be greater than the measured value because the coupling between NH_2_-PEG-S- and ninhydrin or fluorescamine cannot be 100% yield. As such, some of the uncoupled ligands are not counted. These methods are limited to ligands that bear functional groups for coupling with chromophores.

**Figure 5:**
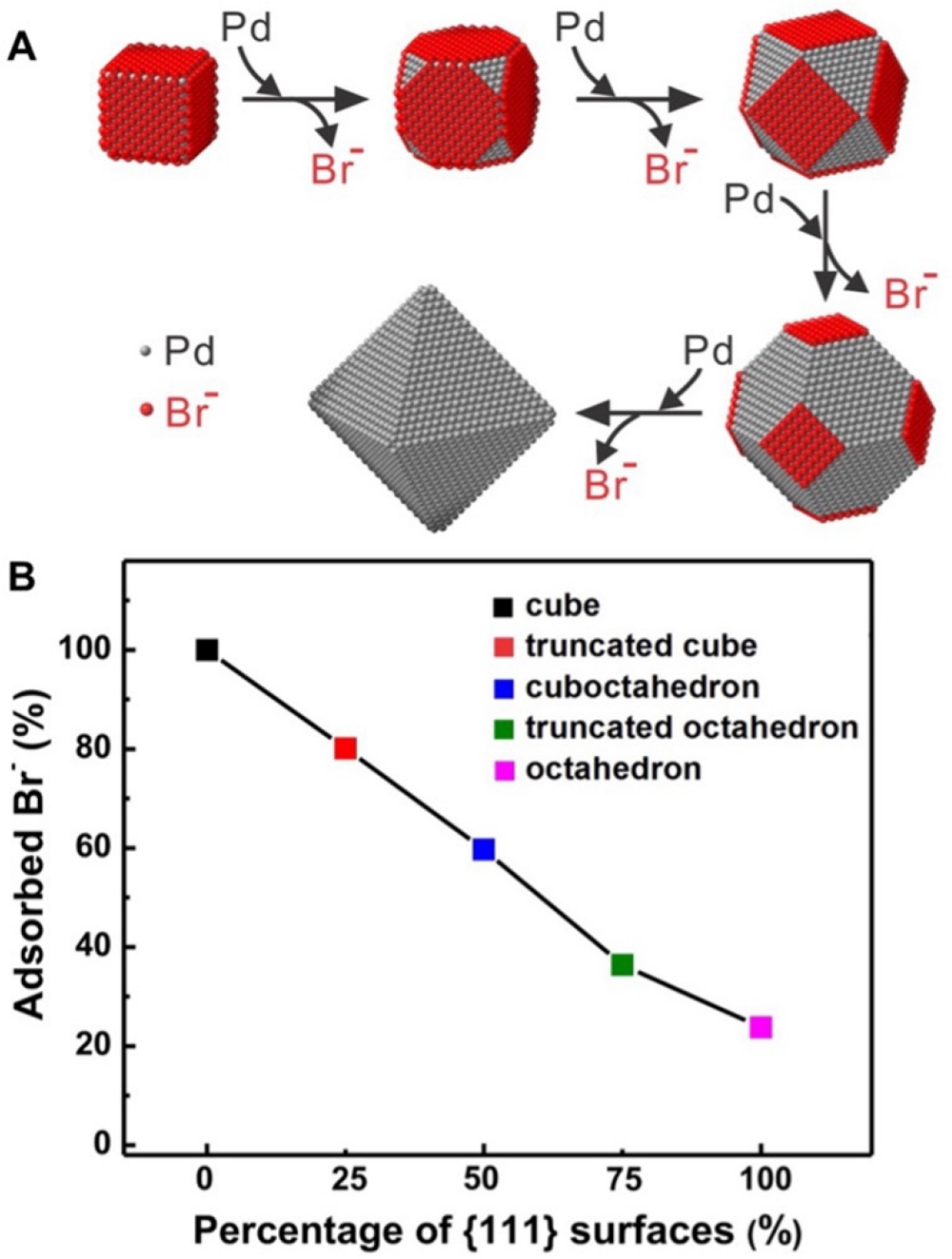
(A) Schematic illustrating the desorption of Br^–^ as Pd cubic seeds grow into octahedra. (B) ICP-MS data showing the continued decrease in residual Br^–^ as growth proceeded. Reproduced with permission from ref (3). Copyright 2013 American Chemical Society.

ICP-MS is a sensitive technique for quantifying the amount of halides adsorbed on the surface of nanocrystals. In one study,^3^ we used ICP-MS to quantify the coverage density of Br^–^ on Pd nanocrystals synthesized using a one-pot method. Specifically, Br^–^ adsorbed onto Pd{100} facets at a coverage density as high as 0.8 ion per surface Pd atom for nanocubes of various edge lengths. Considering the possible truncation at the corners of the Pd nanocubes, the value suggested the formation of a monolayer on the faces. In addition, we tracked the amount of Br^–^ on the surface of Pd cubic seeds as they grew to evolve into an octahedral shape. During the growth, the proportion of {111} facets increased at the expense of {100} facets so that Br^–^ ions kept desorbing from the surface (Figure 5A), which was consistent with the ICP-MS data in Figure 5B. In the end, about 20% of Br^–^ remained on the octahedral nanocrystals, which could be attributed to the remaining {100} facets caused by corner truncation.

**Figure 6:**
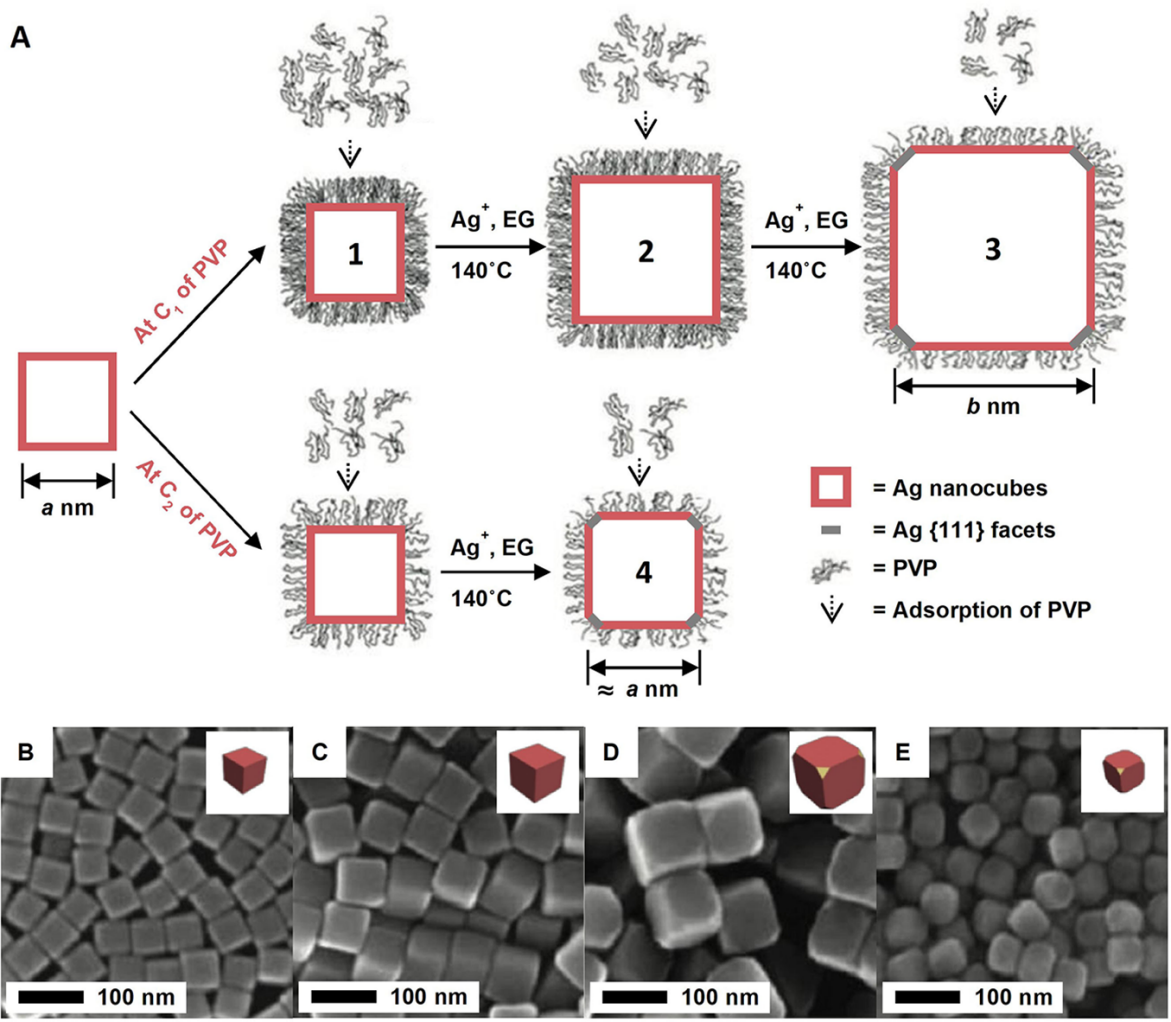
(A) Schematic illustrating the growth of Ag nanocubes in the presence of PVP at a high concentration of *C*_1_ and a critical concentration of *C*_2_. (B–E) SEM images of the corresponding samples 1, 2, 3, and 4 in (A). Reproduced with permission from ref (4). Copyright 2012 American Chemical Society.

We also monitored the shape evolution of nanocrystals during their growth to derive the coverage density of surface ligands.^4^Figure 6A shows Ag nanocubes with an edge length of *a* nm for two growth experiments: (i) at an initial PVP concentration of C_1_ until the edge length increased to *b* where {111} facets started to appear due to insufficient capping of {100} facets (sample 3) and (ii) at a critical initial PVP concentration of C_2_ so that {111} facets started to appear at the beginning (sample 4). Since the coverage densities of PVP (ϕ_PVP_) on Ag{100} facets are the same for samples 3 and 4, it can be derived from the difference in the initial PVP concentrations, *C*_1_ – *C*_2_, between samples 3 and 4:
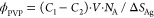
1where *V* is the total volume of the reaction solution, *N*_A_ is Avogadro’s number, and Δ*S*_Ag_ equals the total number of seeds multiplied by 6(*b*^2^ – *a*^2^) nm (i.e., the difference in surface area between the two samples). When 40 nm Ag cubes were used with PVP_55000_ (molecular weight: 55000), the coverage density was 140 repeating units per nm^2^. Since each *N*-vinylpyrrolidone monomer of the PVP_55000_ occupied 0.21 nm^2^, the number of repeating units in each PVP segment folded on the Ag surface was approximately 29. When switching to PVP_10000_, the coverage density increased to 30 repeating units per nm^2^, suggesting that PVP with a lower molecular weight works more effectively in passivating Ag{100} facets.

People have also explored other methods to quantify the amount of PVP adsorbed on the surface of a known number of Ag nanocrystals, including thermogravimetric analysis (TGA) and high-performance liquid chromatography (HPLC).^23^ TGA measured the amount of PVP based on its decomposition and thus mass loss, whereas HPLC was used to obtain the PVP adsorption isotherm by conducting equilibrium adsorption measurements.

## 4 Exchange of Surface Ligand

With the assistance of surface ligands, nanocrystals have been prepared with diverse shapes for various applications.^5^ In many cases, the original ligands might compromise the performance in catalysis or hinder some specific applications like biomedicine due to their intrinsic toxicity and hydrophobicity.^2,24^ As such, the surface ligands must be exchanged, directly or indirectly, for the desired ones to suit the target applications. Examples of direct exchange can be found in the cases of substituting PVP on Ag/Au nanocrystals with thiol-terminated ligands.^16,25^ Specifically, thiol-terminated PEG can quickly replace the PVP on Au nanocages because of the stronger Au–S linkage to make the nanoparticles resistant to protein adsorption and thus long-circulating in bloodstream.^26^ Although the direct method prevails in literature, it has several limitations. First, the new ligand must bind to the surface more strongly than the original ligand. If the difference in affinity is inadequate, it will be difficult to achieve complete substitution. Second, aggregation will occur during the exchange process if the two ligands bear opposite charges.

**Figure 7:**
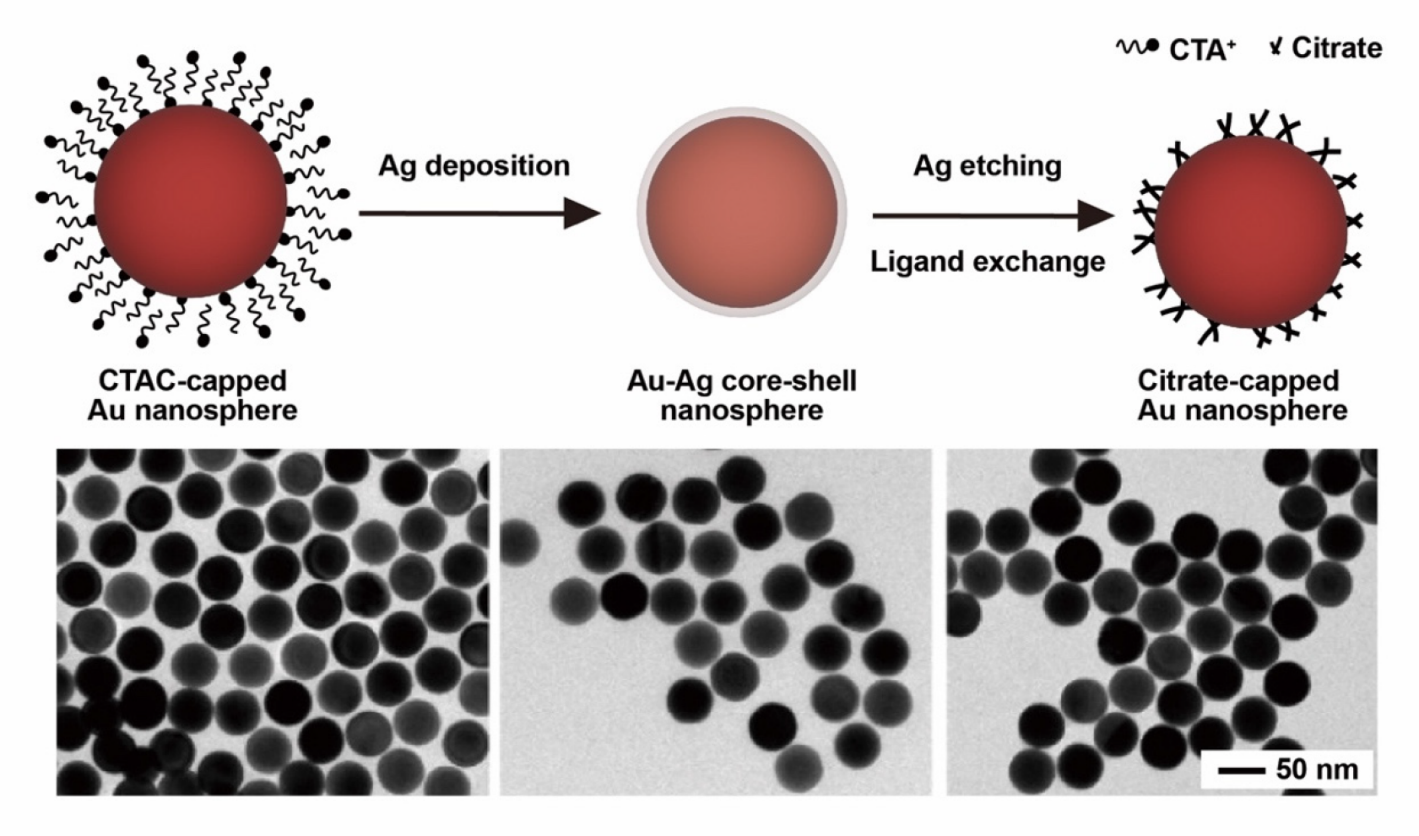
Schematic illustration and TEM images of indirect ligand replacement as assisted by the deposition and etching of Ag. Reproduced with permission from ref (2). Copyright 2018 American Chemical Society.

Alternatively, indirect ligand exchange has been developed by including intermediate steps. In one study, the original ligands were removed by acid treatment under sonication, and the second ligand was then introduced.^27^ This approach tends to trigger aggregation without the adjustment of pH. We developed an indirect method by depositing an ultrathin layer of Ag on Au nanocrystals to effectively remove ligands such as CTAC, followed by etching of the Ag layer while introducing a new ligand (Figure 7).^2^ In this approach, even negatively charged ligands such as citrate can be used without causing aggregation to the positively charged nanocrystals. Furthermore, using this method, the toxic CTAC on Au nanoparticles was exchanged for a biocompatible ligand such as citrate or PVP to augment their biomedical application.

## 5 Removal of Surface Ligand

The presence of surface ligands can compromise the catalytic performance of metal nanocrystals by blocking the active sites. Additionally, the ligand can affect catalytic activity by altering the electronic structure of surface atoms.^28^ It is critical to clean the surface of metal nanocrystals by removing the ligand while maintaining the surface structure. In general, the nanocrystals can be washed with a proper solvent to reduce the coverage density of surface ligand. However, the centrifugal force may cause the nanocrystals to aggregate irreversibly, and it is impossible to remove a ligand that binds strongly to the surface by washing only. Here we compare three methods, where the nanocrystals are deposited on a support in advance to avoid aggregation.

### 5.1 Calcination

Calcination in air can decompose most organic ligands into volatile species such as CO_2_ and H_2_O. In practice, a reductive atmosphere is required to reduce the surface back to the elemental state. In one study, we cleaned the surface of Pd@Pt core–shell octahedral nanocrystals by calcining the sample under an O_2_/N_2_ mixture at 400 °C and then reduced the oxide layer in a H_2_/N_2_ mixture at 100 °C.^29^ Upon calcination, the vibrational peak of CO probe exhibited a blue-shift owing to the removal of PVP from the surface. Otherwise, it would donate electrons to the metal atoms and thus weaken the CO bond. Depending on the temperature and environment involved, calcination often causes morphological and compositional changes to the nanocrystals, in addition to irreversible aggregation or fusion.

### 5.2 Heating in a Solution

**Figure 8:**
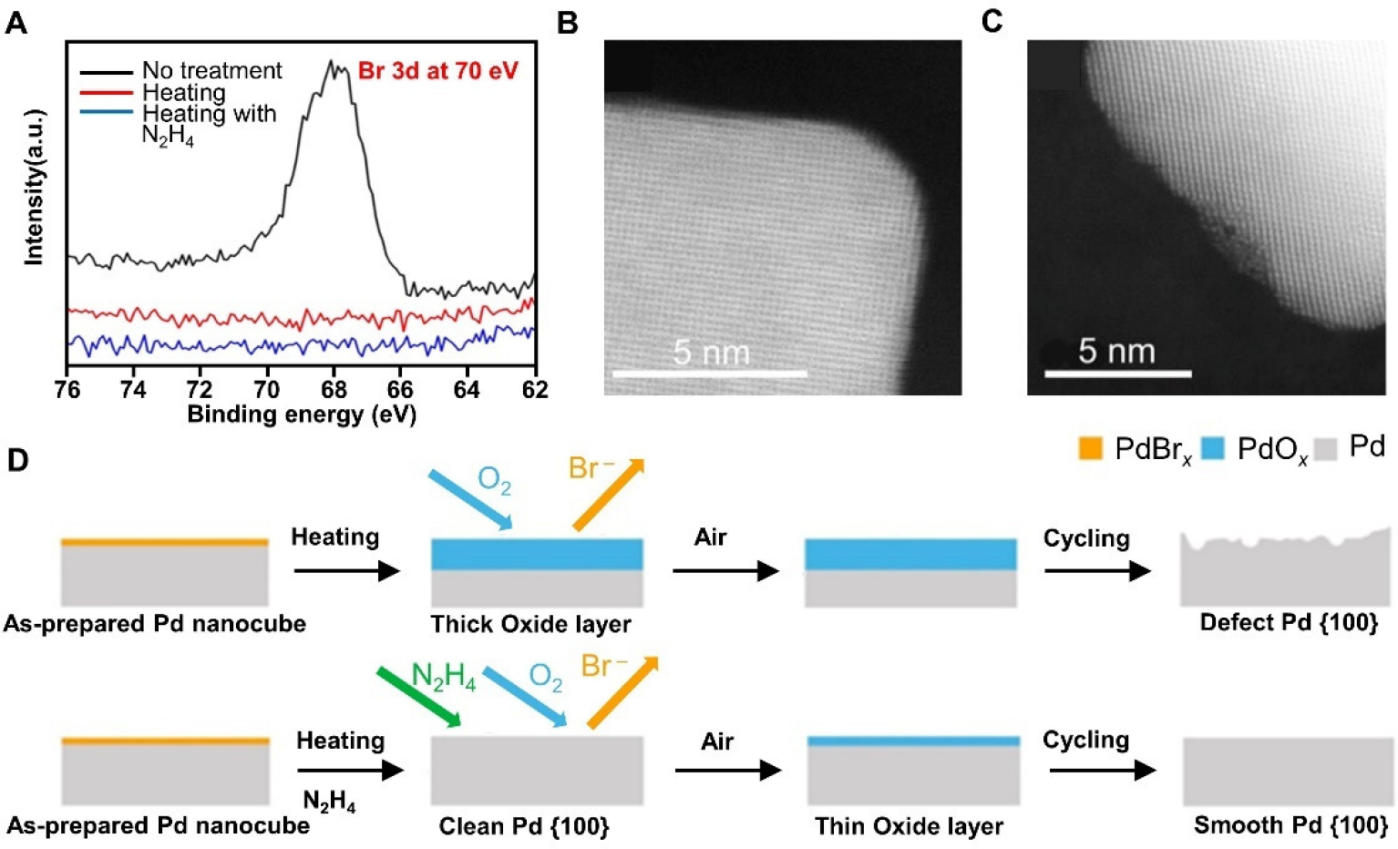
(A) XPS spectra confirming the removal of Br^–^ during heating. (B and C) HRTEM images of Pd nanocubes, heated in the absence and presence of N_2_H_4_, respectively, after potential cycling in HClO_4_. (D) Schematic illustrating why smooth and defected {100} facets were formed when the samples were heated in the presence and absence of N_2_H_4_, respectively. Reproduced with permission from ref (30). Copyright 2020 Wiley-VCH.

Different from calcination, which typically involves heating the sample in a gaseous environment, heating in a solution weakened the binding between the ligand and surface to facilitate desorption. We demonstrated this concept by heating an aqueous suspension of Pd nanocubes to 95 °C to remove the chemisorbed Br^–^.^30^ A relatively thick layer of PdO_*x*_ was formed, which led to significant surface destruction during electrochemical cycling. In addressing this issue, we added a trace amount of N_2_H_4_ into the aqueous suspension of Pd nanocubes to remove the chemisorbed Br^–^ without causing significant oxidation to the surface, as illustrated in Figure 8. Specifically, the Br 3d XPS peak disappeared after heating at 95 °C regardless of the presence/absence of N_2_H_4_, suggesting the removal of chemisorbed Br^–^. When a trace amount of N_2_H_4_ was added, the {100} facets were well preserved. In contrast, atomic steps and trenches appeared on the surface in the absence of N_2_H_4_. In addition, the contrast difference in Figure 8C suggested that the atoms in the trench did not have well-defined lattice spacing or atom arrangement and hence were in a disordered form. The oxide layer was found to be 1.1 and 0.43 nm thick when the treatment was conducted in the absence and presence of N_2_H_4_, respectively.

Typically, Br^–^ withdraws electrons from metal atoms upon chemisorption, so the surface is partially oxidized. Introducing a reductant such as N_2_H_4_ brings the surface atoms back to the elemental state. As such, the binding between the ligand and surface atoms would be weakened, facilitating ligand removal. We also demonstrated this concept in other studies,^18,31^ where Pd nanocubes were heated in ethylene glycol to remove the chemisorbed Br^–^. According to XPS, the intensity of the Br 3d peak disappeared, implying their successful removal. Altogether, heating the nanocrystals in a solution offers an effective way to remove the ligand by weakening the interaction between the ligand and surface atoms.

### 5.3 UV-ozone Treatment

UV-ozone treatment can remove the ligand at room temperature, helping preserve the composition and surface structure of the nanocrystals. It typically involves a series of chemical reactions. First, the ligand molecules are excited upon the absorption of UV light. Meanwhile, O_2_ from the air absorbs UV light to dissociate into atomic oxygen and ozone, which can further dissociate into atomic oxygen. Subsequently, the excited ligand reacts with atomic oxygen to form volatile species such as CO and CO_2_.^32^ We removed PVP from Pd nanocubes by UV-ozone treatment.^33^ A UV source with outputs at 185 and 257 nm was used, with the former dissociating O_2_ and the latter exciting PVP. After treatment, we noticed a drastic reduction in the N 1s XPS peak. Additionally, the FT-IR peak corresponding to the C–H stretching of PVP dropped in intensity. These results confirmed the removal of PVP. Most importantly, the Pd nanocubes retained their size and shape distributions. This method has several drawbacks: (i) CO has a high affinity toward most precious metals and may cause surface poisoning; (ii) UV-ozone treatment may increase the acidity of the support and compromise the durability of the catalyst toward some reactions; and (iii) the ligand adsorbed on regions that cannot be directly irradiated by the UV light will stay. Therefore, the protocol always needs optimization depending on the metal/ligand involved and the application.

To conclude, the ligands adsorbed on metal nanocrystals can be removed by calcination, heating in a solution, and UV-ozone treatment. Other methods have also been reported, such as holding the nanocrystals at an electrochemical potential^34,35^ and plasma etching.^36^

## 6 Applications

In addition to their vital roles in the colloidal synthesis of nanocrystals, surface ligands are extensively explored for postsynthesis modification to meet the requirements from various applications. To this end, Au nanocrystals have been modified with thiol-based ligands for biomedical applications because of their unique optical properties and the robustness of Au-thiolate chemistry.^37^ Furthermore, by introducing a proper ligand to a specific set of facets, colloidal nanocrystals can be directed to assemble into complex structures or superlattices.^38^

### 6.1 Nuclear Imaging and Nanomedicine

**Figure 9:**
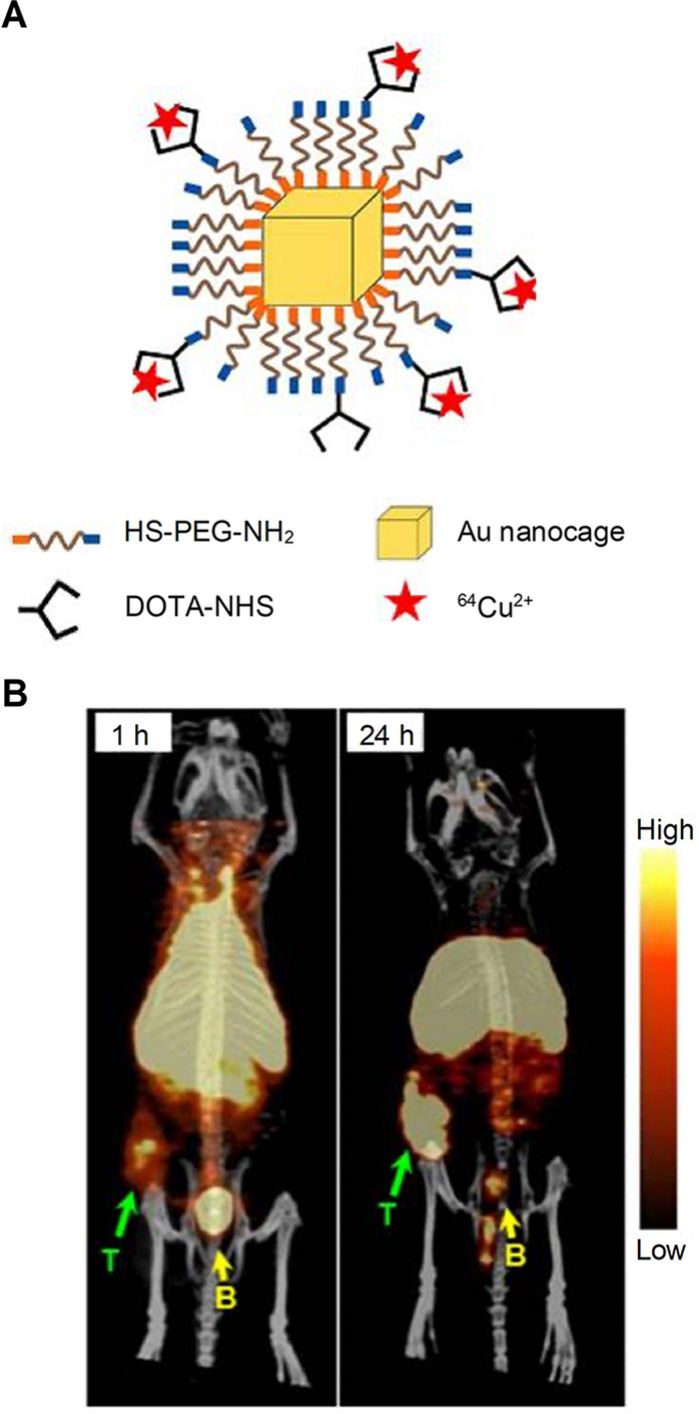
(A) Schematic illustration of ^64^Cu-DOTA-PEG-Au nanocages for PET. (B) PET images of 30 nm ^64^Cu -DOTA-PEG-Au nanocages in a mouse bearing an EMT-6 tumor at 1 and 24 h post-injection. Reproduced with permission from ref (26). Copyright 2012 American Chemical Society.

Positron emission tomography (PET) is one of the commonly used techniques for early stage cancer diagnosis because it is highly sensitive and noninvasive. In the research setting, it is used to track the biodistribution of a nanomedicine and thus enable image-guided therapy. To this end, it is critical to label the nanomedicine with a radioisotope and thus enable tracking by PET. Figure 9A shows an example, where the surface of Au nanocages was conjugated with NH_2_-PEG-S- and then labeled with radioactive ^64^Cu^2+^ for PET.^37^ Specifically, 30 nm Au nanocages were synthesized and subsequently incubated with bifunctional NH_2_-PEG-SH to allow the formation of Au–S bond. The amine group at the distal end was then coupled with 1,4,7,10-tetra-azacyclododecane-1,4,7,10-tetraacetic acid mono(*N*-hydroxy succinimide ester) (DOTA-NHS-ester) through an amide linkage. Finally, radioactive ^64^Cu^2+^ was introduced through chelation with DOTA. As shown in Figure 9B, the Au nanocages were substantially accumulated inside the tumor due to their long blood circulation time and outstanding enhanced permeability and retention (EPR) effect. In this study, the bifunctional NH_2_-PEG-SH ligand plays an essential role by serving as a bridge between Au nanocages and ^64^Cu^2+^. While the thiol group binds strongly to the Au surface, the primary amine allows for the conjugation of DOTA and thus incorporation of radioactive ^64^Cu^2+^ for PET. These functional groups hold the key to robust radiolabeling and reliable PET.

### 6.2 Tumor Targeting

**Figure 10:**
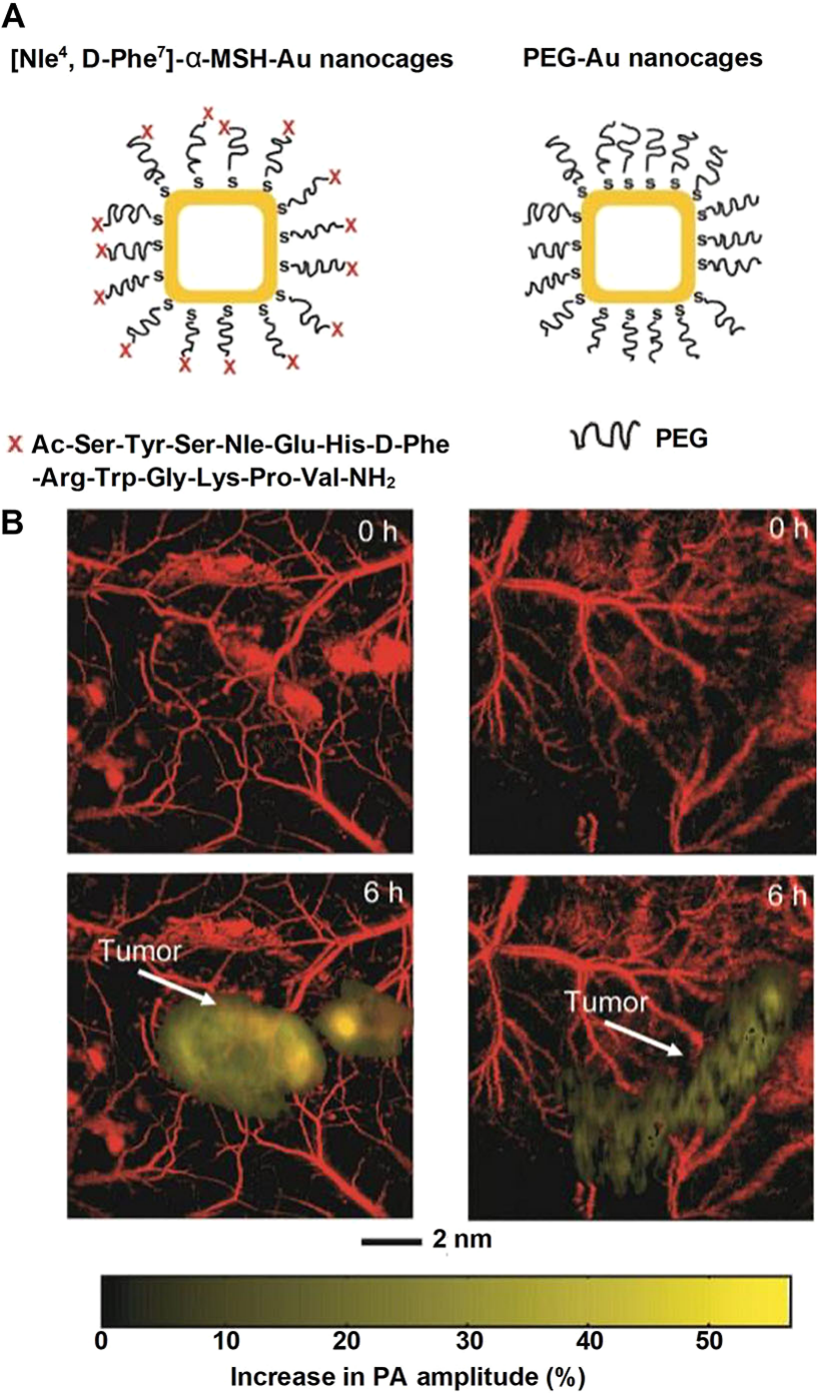
(A) [Nle^4^, d-Phe^7^]-α-MSH-Au nanocages (left) for active targeting of melanoma, with the PEG-covered nanocages (right) as a control group. (B) PAT time-course coronal MAP images of B16 melanoma targeted by different nanocages before injection and 6 h post-injection. Reproduced with permission from ref (39). Copyright 2010 American Chemical Society.

The EPR effect of nanoparticles has been widely explored as a passive targeting mechanism for the development of advanced cancer therapeutics. To better utilize the biochemical properties of cells to be targeted, ligands such as peptides, antibodies and antibody fragments, and nucleic acids (e.g., aptamers) can be added to enhance the targeting efficiency. This new targeting mode based on molecular recognition is known as active targeting, where the ligand–receptor binding allows the nanoparticle to selectively and strongly bind to the surface of a specific type of cells. This strategy has proven to be effective *in vitro* and, to a certain extent, *in vivo*. In one study,^39^ we used photoacoustic tomography (PAT) to compare the melanoma-targeting capabilities of Au nanocages with different surface ligands. Specifically, the surface of Au nanocages was conjugated with PEG chains terminated in [Nle^4^, d-Phe^7^]-α-melanocyte-stimulating hormone ([Nle^4^, d-Phe^7^]-α-MSH, Figure 10A, left) and PEG chains (Figure 10A, right) for active and passive targeting, respectively. Figure 10B shows the PAT images recorded at different time points after the injection of the nanocages. At *t* = 6 h post-injection, the signal (golden color) from the [Nle^4^, d-Phe^7^]-α-MSH Au nanocages increased by 36%, whereas the control group only increased by 14%. The enhanced accumulation of [Nle^4^, d-Phe^7^]-α-MSH Au nanocages arose from the strong binding to the α-MSH receptor overexpressed on the surface of melanoma cells. A parallel *in vitro* study also confirmed the higher uptake of [Nle^4^, d-Phe^7^]-α-MSH Au nanocages by melanoma cells. On average, each melanoma cell was able to uptake 123 [Nle^4^, d-Phe^7^]-α-MSH Au nanocages, which was 3.5 times as high as the PEG-covered nanocages.

### 6.3 Self-Assembly

**Figure 11:**
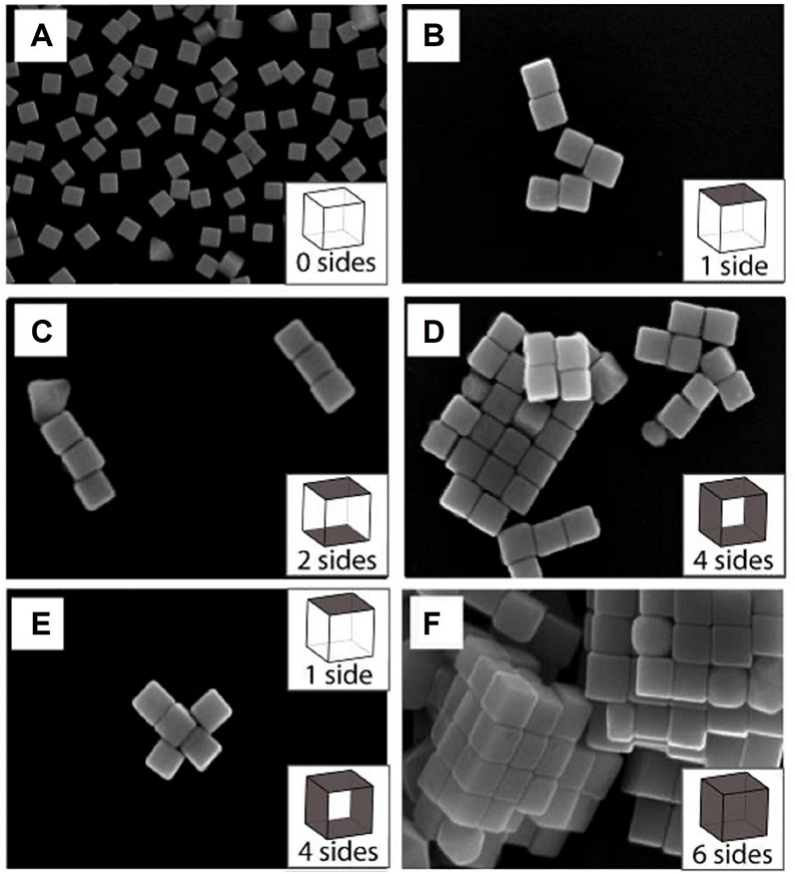
SEM images of structures assembled from Ag nanocubes with (A) zero, (B) one, (C) two, (D) four, (E) one plus four, and (F) six of the faces functionalized with ODT. Reproduced with permission from ref (38). Copyright 2008 Wiley-VCH.

Self-assembly offers a simple and versatile route to complex structures by coding the surface of building blocks with specific interactions. The assembly of metal nanocrystals can be directed by derivatizing their surface with proper ligands. Figure 11 shows an example where the faces of Ag nanocubes were functionalized, in different patterns, with a hydrophobic monolayer based upon octadecanethiol (ODT).^38^ When suspended in water, the Ag nanocubes preferred to come into contact through their hydrophobic faces to minimize their exposure to water. One of the faces can be selectively functionalized by depositing the nanocubes on a Si substrate (Figure 11A), followed by immersion in an ethanolic solution of mercaptohexadecanoic acid. As such, the face protected by the Si substrate could be functionalized by ODT later. As illustrated in Figure 11B, the Ag nanocubes tended to dimerize if one of the faces was functionalized with ODT. Additionally, linear chains and two-dimensional rafts, respectively, were formed when two and four of the faces were functionalized with ODT (Figure 11C,D). If four or more of the side faces were functionalized, three-dimensional lattices would be formed. This work demonstrates that the assembly of nanocrystals can be manipulated by modifying their surface with a proper ligand.

## 7 Conclusion and Perspectives

This Account summarizes our research endeavors in developing methods for quantifying, exchanging, and removing surface ligands on noble-metal nanocrystals. Methods including SERS, FT-IR, XPS, and EDX, can be used to obtain qualitative information about the surface ligand. Meanwhile UV–vis and ICP-MS provide a quantitative measure of the coverage density of certain types of surface ligands. In some cases, it is feasible to derive the coverage density of the ligand by tracking the morphological changes of metal nanocrystals during their overgrowth in the presence of a specific amount of the ligand. We also discuss direct and indirect methods for substituting the ligand molecules. Furthermore, we compare three methods, including calcination, heating in water containing a trace amount of N_2_H_4_, and UV-ozone treatment at room temperature, for removing ligands from the surface of nanocrystals. Finally, we use three examples to illustrate the multifaceted roles of surface ligands in enabling or enhancing the use of nanocrystals in nanomedicine, tumor targeting, and self-assembly.

Despite the incredible progress, there are still a number of scientific issues or technological challenges that need to be addressed before we can push surface ligands and metal nanocrystals to the next level of success. One of the major challenges is to identify suitable surface ligands for the syntheses of bi- and multimetallic nanocrystals with specific morphologies. As reported in literature,^5,19,40^ the same surface ligand may stabilize different types of facets in the case of alloys. For example, CO is an effective capping ligand for the synthesis of Pt nanocubes by selectively capping the {100} facets. When applied to Pt–Ni bimetallic nanocrystals, however, {111} facets are selectively capped for the production of an octahedral shape. Another challenge is still the precise quantification of surface ligands. Various techniques are reviewed here and in another article.^41^ In general, we still do not have a universal method that is applicable to all types of surface ligands. Many of the techniques can only be used for qualitative measurements without involving extensive approximation and/or simulation. It is hoped that this Account will inspire the readers to further develop more advanced tools for this characterization task.
